# The Nigger in the Woodpile

**Published:** 1898-02

**Authors:** 


THE NIGGER IN THE WOODPILE.—The Gallagher bill against vivisection is being pushed in the Senate with increased vigor and venom, and, unless there is strong and immediate counteraction, it will pass. The JOURNAL is pleased to learn that a strong committee, appointed by the Congress of American Physicians, is actively at work in a way that is calculated to have a telling effect towards beating it. We have been at a loss to understand the animus of this bill heretofore; we did not believe the "Humane Society" people, who are charged with getting it up, were such fools as not to listen to reason. We have found out now, why it is: Gallagher, the author of the infamous, fool measure, is a *homeopathic doctor*. That accounts for the venom and the absurdity of the measure; the implacable determination to get it through in spite of the thousands of reasons why it should not pass. It is their same old game—fighting the medical profession; it is ignorance and bigotry. Homeopaths do not study physiology, no need to, and they don't want anybody else to do so. What need of knowledge to give sugar of milk and moonshine? Bah! That otherwise sensible people will patronize a homeopath, with his infinitesimal doses of inert stuff, is to be accounted for only in one way: it is a fad in some cities, and the parvenues ape the few who set the example. Gallahger is determined to "*make* her go," not "*let* her go." He should be choked off.

